# Identifying high-risk areas of bacillary dysentery and associated meteorological factors in Wuhan, China

**DOI:** 10.1038/srep03239

**Published:** 2013-11-21

**Authors:** Zhenjun Li, Ligui Wang, Weige Sun, Xuexin Hou, Haiyan Yang, Lina Sun, Shuai Xu, Qiangzheng Sun, Jingshan Zhang, Hongbin Song, Hualiang Lin

**Affiliations:** 1 State Key Laboratory for Infectious Disease Prevention and Control, National Institute for Communicable Disease Control and Prevention, Chinese Center for Disease Control and Prevention; Collaborative Innovation Center for Diagnosis and Treatment of Infectious Diseases; 2 Institution of Disease Control and Prevention, Academy of Military Medical Sciences; 3 College of Public Health, Zhengzhou university, Zhengzhou, China; 4 Guangdong Provincial Institute of Public Health, Guangdong Provincial Center for Disease Control and Prevention, Guangzhou, Guangdong, China; 5 These authors contributed equally to this work.

## Abstract

Spatial distribution of bacillary dysentery incidence was mapped at the district level in Wuhan, China. And a generalized additive time series model was used to examine the effect of daily weather factors on bacillary dysentery in the high-risk areas, after controlling for potential confounding factors. Central districts were found to be the high-risk areas. The time series analysis found an acute effect of meteorological factors on bacillary dysentery occurrence. A positive association was found for mean temperature (excess risk (ER) for 1°C increase being 0.94% (95% confidence interval (CI): 0.46% to 1.43% on the lag day 2), while a negative effect was observed for relative humidity and rainfall, the ER for 1% increase in relative humidity was −0.21% (95% CI: −0.34% to −0.08%), and the ER for 1 mm increase in rainfall was −0.23% (95% CI: −0.37% to −0.09%). This study suggests that bacillary dysentery prevention and control strategy should consider local weather variations.

Bacillary dysentery is a bacterial infection of the mucosal surface of the intestines caused by Shigella. Patients who are infected with Shigella often develop bloody diarrhea, fever, and stomach cramps with an incubation period of one or two days^1^. The infection can be transmitted by the fecal-oral route via contaminated water, food, or person-to-person contacts. It remains a public health problem in both developed and developing countries^2^,^3^. In China, a considerable disease burden still exists, particularly among children and the elderly, although the morbidity and mortality have decreased considerably in the last decade^4^. According to the National Notifiable Diseases of China, there were about 269,299 bacillary dysentery cases reported in 2009, with an incidence rate of 20.28 per 100,000^5^. It is the third leading notifiable disease, next only to Tuberculosis and Hepatitis B in China^5^.

The incidence of bacillary dysentery varied considerably from place to place in China, with the highest incidence of 142.78 per 100,000 in Beijing, and lowest incidence being reported in Jiangsu and Guangdong Provinces^5^. The incidence of bacillary dysentery has been reported to exhibit distinct seasonality in a number of areas. For example, summer and autumn peak was observed in Jinan, Shenyang, Shanghai and Huzhou^2^,^6^,^7^,^8^,^9^. Epidemic peaks were observed in September-November in Dhaka, Bangladesh^10^. The seasonality of bacillary dysentery incidence indicated that meteorological factors might play an important role in its epidemiology, which has gained increasing concerns in recent years, mainly due to the global climate change^6^,^7^. Ambient temperature may directly influence the replication and survival of the pathogens in the environment. Rainfall, especially heavy rainfall events, may affect the frequency and level of contamination of drinking water. Weather variations may also affect population behavior, including eating habits, which may indirectly influence the transmission of this infection^2^. Several studies have investigated the association between diarrheal diseases and meteorological factors^2^,^11^. For example, a time series analysis in Jinan found that a 1°C increase in maximum temperature might relate to about 11.40% (95% confidence interval: 10.19%–12.69%) increase in bacillary dysentery^2^. Using a Hockey Stick model, a study found that the thresholds for the effects of maximum and minimum temperatures were 17°C and 8°C in a northern Chinese city, but no thresholds were detected in the southern city^12^. In Shenyang, ambient temperature, precipitation and relative humidity were found to be positively associated with monthly incidence of bacillary dysentery^7^. A large scale study observed a strong association between extreme precipitation and water-borne infectious disease outbreaks with 2-month lag in the United States^11^. These studies mainly used monthly data to detect the association between temperatures and dysentery transmission, however, more finer data, such as daily or weekly data, could make the estimation more accurate.

Wuhan City in Hubei Province is the largest mega-city in Central China and in the middle reaches of Yangtze River (Figure 1). It had a relatively high prevalence of bacillary dysentery in recent years, and the incidences among the counties within Wuhan City were not homogenous. A better understanding of the spatial pattern of bacillary dysentery incidence would help identify high-risk areas and might help appropriate allocation of health care resources for better disease control and prevention. Moreover, no study has been conducted to examine the effect of meteorological factors on the occurrence of bacillary dysentery in this city.

The present study, using existing surveillance data, described the spatial distribution of bacillary dysentery incidence at the district level in Wuhan, and quantified the association between daily meteorological factors (including daily mean temperature, relative humidity, rainfall, and wind speed) and bacillary dysentery occurrence in high-risk areas of Wuhan.

## Results

**Figure 2:**
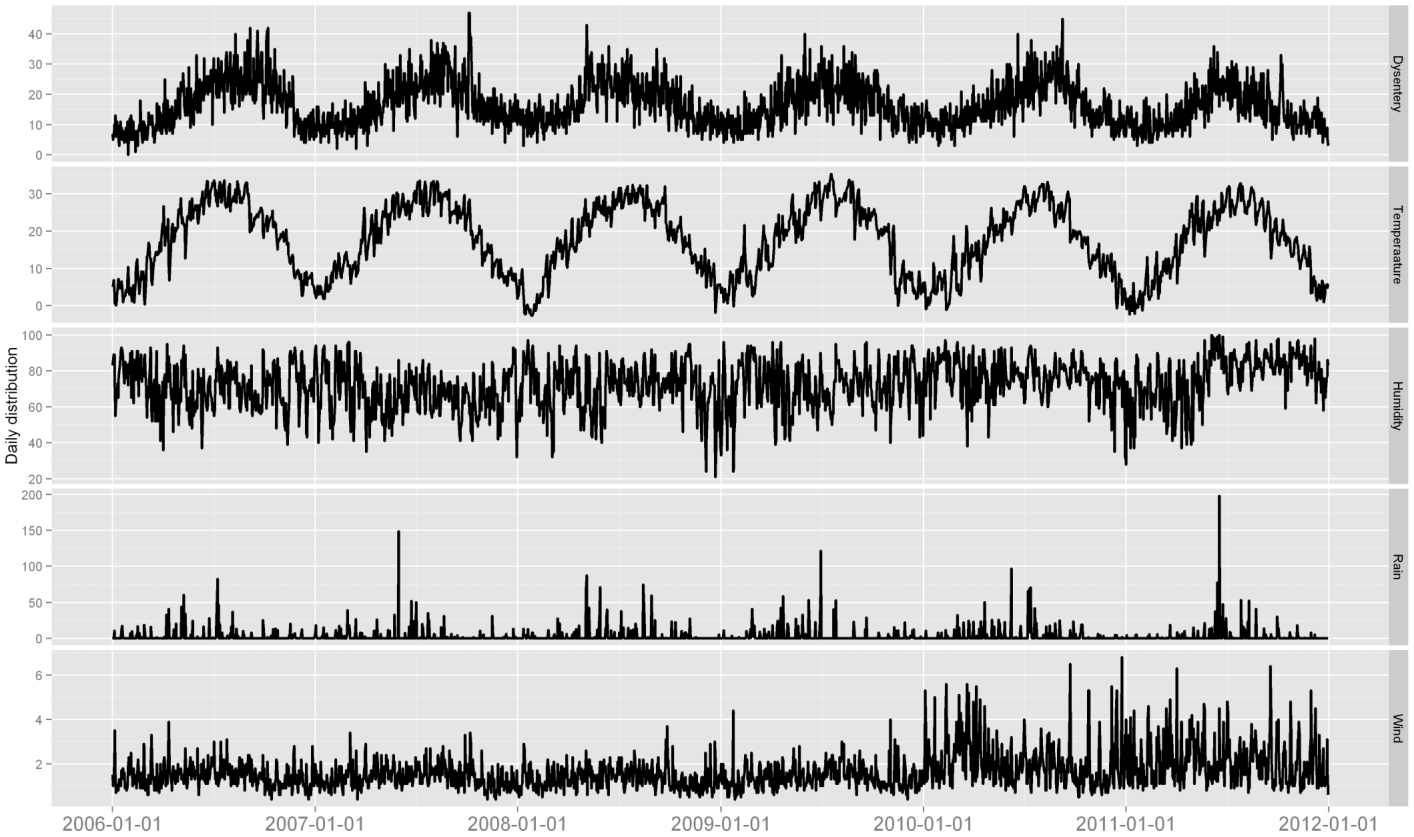
The time series of daily bacillary dysentery and meteorological variables in Wuhan, 2006–2011 (unit: Temperature (°C), relative humidity (%), rainfall (mm), and wind speed (m/s)).

Between 1 January 2006 and 31 December 2011, a total of 36,487 bacillary dysentery cases were reported in Wuhan City. The annual cases ranged from 5,418 to 6,407, with a mean of 6,081 cases per year. The annual incidence ranged from 68.9 per 100,000 to 81.5 per 100,000 population, with a mean of 77.4 per 100,000. There were more male cases with a male-to-female sex ratio of 1.3:1 (20,422:16,065). The descriptive statistics for weather factors and bacillary dysentery were shown in Table 1. The mean level of daily mean temperature, relative humidity, rainfall and wind speed in Wuhan were 17.6°C, 72.3%, 3.1 mm and 16.4 m/s, respectively. Figure 2 showed the time series of daily weather variables and bacillary dysentery count in Wuhan. There were seasonal pattern in these factors. Summer epidemic peak was observed the occurrence of bacillary dysentery, with the highest risk occurring in June-September months.

**Figure 3:**
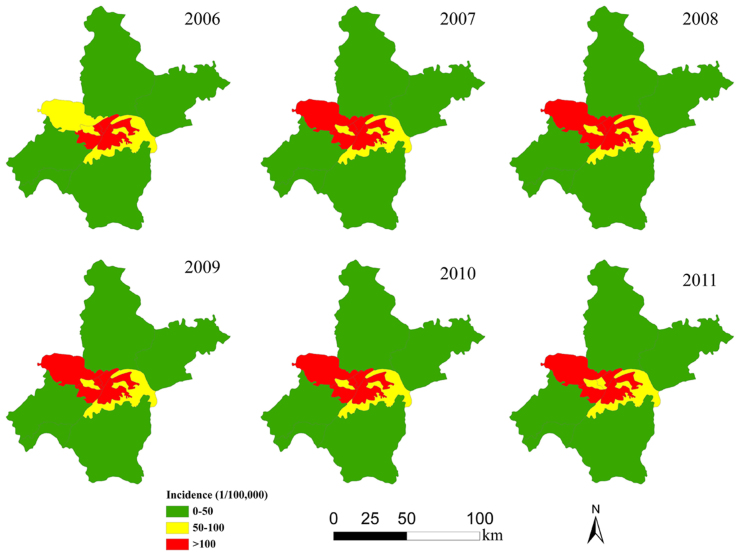
Annual average incidence of bacillary dysentery among the districts in Wuhan, China, 2006–2011 (created with ArcGIS software, 9.0).

Figure 3 showed the spatial distribution of annual incidence of bacillary dysentery over the 6 years study period. Central districts were found to be the high-epidemic areas of bacillary dysentery occurrence, which was quite consistent across the 6 years. The further time-series analysis was conducted in high-risk areas with 5 clustering districts.

**Figure 4:**
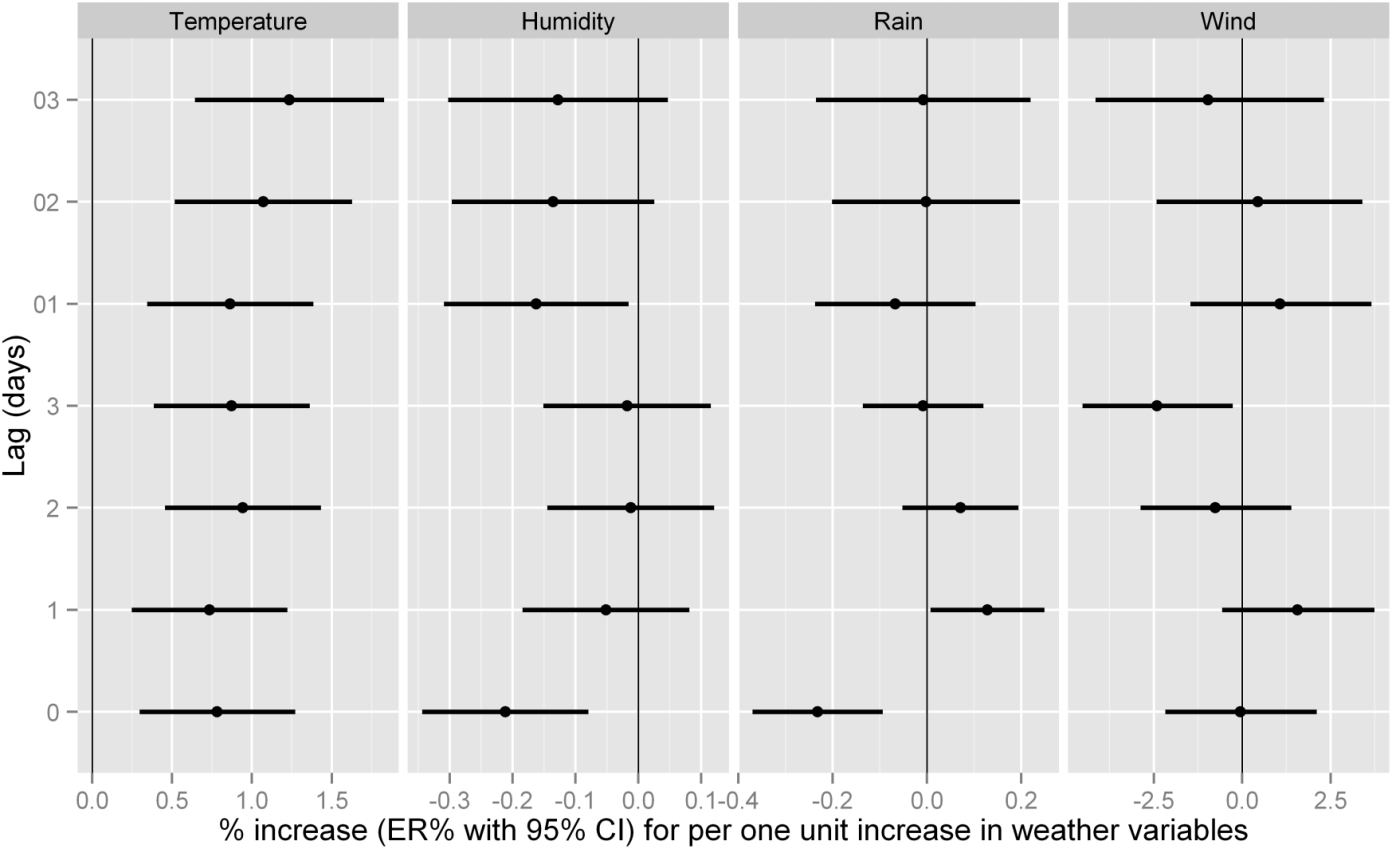
Excessive risk (ER with 95% CI) in bacillary dysentery in Wuhan for one unit increase in daily meteorological factors for the current day (lag0) to 3 days before the current day (lag 3) and moving average (lag 01, 02 and 03).

The association between various meteorological factors and bacillary dysentery was presented in Figure 4. A statistically significant association was detected for temperature (at lag days 0–3, lag01 and lag03), relative humidity (at the current day and lag01), and rainfall (at the current day), while no significant association was detected for wind speed along the lag days. A positive association was found between mean temperature and bacillary dysentery, the ER for a 1°C increase was 0.78% (95% CI: 0.30% to 1.27%) on the current day, 0.94% (95% CI: 0.46% to 1.43%) on the lag day 2, respectively. Negative association was found for relative humidity and rainfall on the current day, the ER for 1% increase in relative humidity was −0.21% (95% CI: −0.34% to −0.08%), and the ER for 1 mm increase in daily rainfall was −0.23% (95% CI: −0.37% to −0.09%).

In the analysis of moving average of the weather variables, significant association was also detected for temperature and relative humidity (Figure 4). The ER for 1°C increase in temperature was 0.86% (95% CI: 0.34% to 1.38%) on lag01, and 1.23% (95% CI: 0.64% to 1.83%) on lag03, respectively. For relative humidity, the ER for 1% increase on lag01 was −0.16% (95% CI: −0.31% to −0.02%).

**Figure 5:**
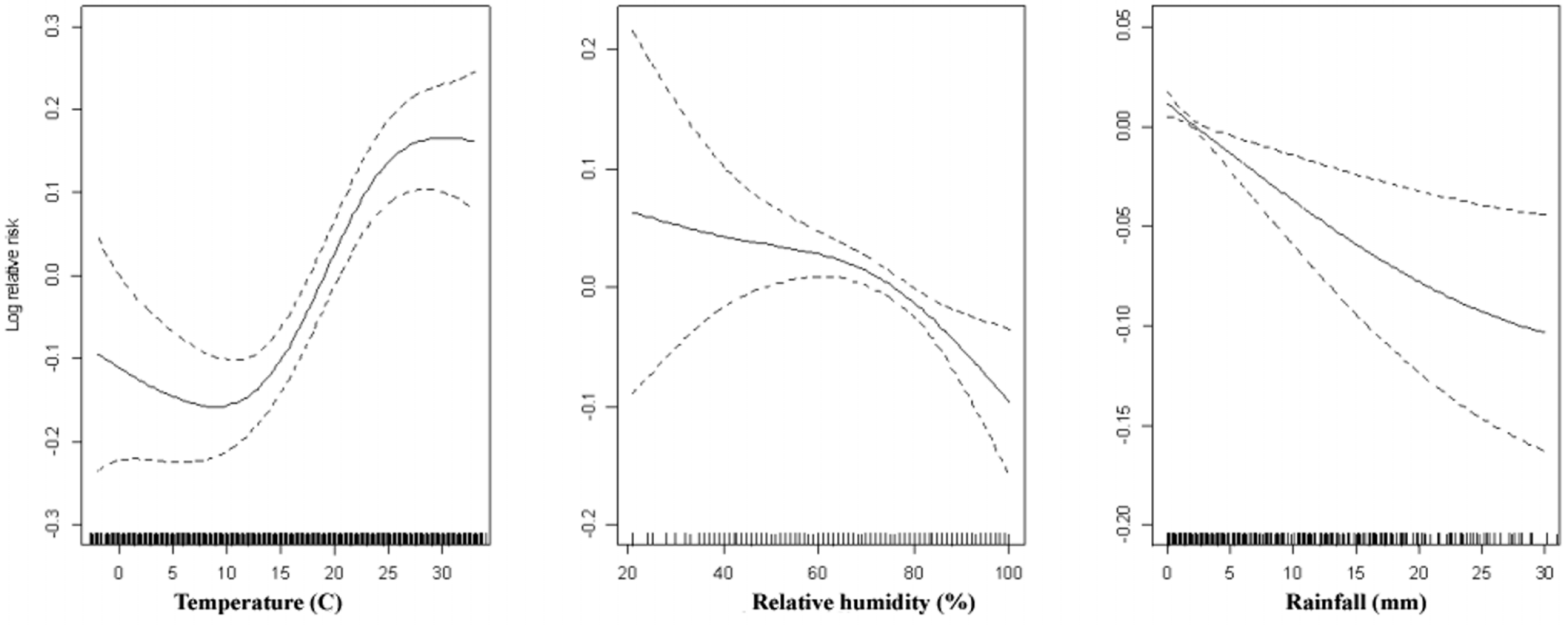
Smoothing plots of daily mean temperature, relative humidity and rainfall against bacillary dysentery in Wuhan. Confounding factors included time trend, day of week and public holidays.

The dose-response relationship for the three meteorological variables with bacillary dysentery occurrence was illustrated in Figure 5. An almost linear relationship was observed for the three meteorological variables.

In the sensitivity analysis, we changed the df (5, 6, 8) for calendar time to control for seasonality and long-term trend, which produced similar results. We changed the df (4–7) for meteorological factors, the estimated effects of temperature, relative humidity and rainfall were not substantially changed. We also did the time series analysis in the low-risk areas, and found a similar result as that in high-risk areas (Figure s1), briefly, we found a positive effect of temperature on the current day (ER = 0.68%, 95% CI: 0.09% to 1.27%), and a negative effect of relative humidity on lag0 (ER = −0.15%, 95% CI: −0.28% to −0.02%).

## Discussion

Diarrhea and other water-borne infectious diseases in developing countries having been rising over the past years largely due to unsafe water, inadequate sanitation and poor hygiene. This study examined the geographic distribution of bacillary dysentery incidence in Wuhan City, and examined the association between the occurrence of bacillary dysentery and meteorological variables in the high-risk areas of this city. Central areas in Wuhan City were found to have relatively higher risk of bacillary dysentery. Temperature, relative humidity and rainfall were found to be associated with the occurrence of this disease.

Climate change has already caused and will continue to bring challenges to public health and communicable disease control and prevention. The effects of meteorological factors on transmission of enteric infection have raised less attention compared with vector-borne diseases, e.g., malaria, dengue fever, and hemorrhagic fever with renal syndrome^13^,^14^,^15^. The current study showed that weather variables were also significantly associated with the risk of bacillary dysentery.

The transmission of Shigella could be affected by many factors, including people's dietary pattern, hygiene behavior, susceptibility to different pathogen strains, and sensitivity to the available drugs as well as local weather conditions. Weather variability that impacts the incubation and survival of Shigella in the environment is considered as one of the major environmental predictors to the risk of Shigella transmission^7^.

A few studies have reported that temperature was the key weather variable in the transmission of bacillary dysentery, finding of the present study was consistent with previous reports. In China, for instance, a study found that 1°C increase in maximum or minimum temperature was associated with 12% increase in bacillary dysentery in Jinan City and 16% increase in Shenzhen^12^. In Peru, for instance, each 1°C increase in temperature was associated with an 8% increase in the risk of severe children diarrhea^16^. Higher temperature may increase exposure to the pathogens, promote the growth of the bacteria, and lengthen the survival of the bacteria in the environment and contaminated food^16^. It was also possible that high temperature may be associated with the behavioral pattern of the population, such as increased demand for water and less conscientious healthy activities, which could facilitate the transmission of this disease^17^. In the context of global climate change, it has been projected that the temperature will continue to rise in the future^18^, more specific interventions should be considered at this stage to adapt and mitigate the possible impacts on the transmission of bacillary dysentery.

Some study^2^ has suggested that there is threshold in the association between temperature and bacillary dysentery, above which, the relationship was linear, which is generally consistent with our finding as shown in the dose-response curve in Figure 5. Lagged effects of temperature on enteric infections have been reported in previous studies, ranging from several days to weeks^2^,^19^. In this study, we found a relatively acute effect of temperature on bacillary dysentery, which is more biological plausible due to the short incubation period of this disease^1^. This discrepancy with previous studies might be due to the observation scales. This study was based on daily observations, while previous analyses usually used monthly or yearly data^2^,^12^,^19^. So we suggested to use more finer scale data in future endeavors to examine the effect of weather variables on the transmission of bacillary dysentery.

This study found that relative humidity and rainfall was inversely associated with risk of bacillary dysentery in Wuhan, which was inconsistent with our expectation. The underlying reason might be that during the period with high relative humidity and rainfall, the contacts with contaminated food or water were reduced. Few studies have examined the effects of humidity and rainfall on occurrence of bacillary dysentery with inconsistent findings. One study found that shortage of rainfall in the dry season increased the prevalence of diarrheal diseases across Sub-Saharan Africa^20^. While, one study in northeast China observed a positive association for relative humidity and rainfall with bacillary dysentery^7^, a study in Fiji also found a positive association between diarrhea and extremes of rainfall^19^, similar findings were also reported in Taiwan^21^, US^22^, Bangladesh^23^. However, studies in two southern and northern Chinese cities did not detected significant effect of relative humidity and rainfall^2^,^12^. This discrepancy might be due to the difference in socioeconomic status, such as water and sanitation infrastructure in different regions, as well as human hygiene behaviors and population characteristics.

The results of the study could help to understand the seasonality of this disease. During the high epidemic months (June-September), daily mean temperature was around 25.4°C, which was statistically higher than that in other months (13.6°C). However, the daily mean relative humidity was similar, 73.7% and 71.5% in peak months and other months, respectively, and rainfall was also similar, with 3.3 mm in high risk months and 3.0 mm in low risk months. These comparison indicated that temperature may play an important role in the seasonality of bacillary dysentery.

Our study had two major strengths. Firstly, this study investigated the impacts of local meteorological factors on bacillary dysentery at a daily scale in the high-risk area of Wuhan City using a time series analysis approach. The model can flexibly examine the possible relationship of day-to-day variation of weather conditions on day-to-day variation in the disease occurrence^24^. Although this model had many parameter specifications, our sensitivity analyses suggested that the results of the study were insensitive to the model specification. Secondly, our study suggested that temperature might be the key factor for the seasonality of the occurrence of bacillary dysentery, which has not been reported in previous studies. Meanwhile, a few limitations should be noted when interpreting findings from this study. Our study was ecological in study design, which did not allow us to explore individual-based association and limited our capacity of causal inference^24^. Also due to the lack of individual data and detailed clinical information, we were unable to further examine the association in more details. Our analysis was preliminary and exploratory, therefore, we could not exclude the possibility of a spurious finding or unmeasured confounding factors that might be associated with both weather variables and bacillary dysentery occurrence.

In conclusion, ambient temperature, relative humidity and rainfall might be most contributing weather predictors of the transmission of bacillary dysentery in Wuhan. Bacillary dysentery prevention and control strategy should consider these meteorological factors.

## Methods

As the capital city of Hubei Province, Wuhan is an important transport center in China with a total population of about 9.7 million over an area of 2,000 km^2^. The climate in this city is a subtropical humid monsoon, with sufficient rainfall.

Bacillary dysentery is a national legally notifiable infectious disease in China, all clinical and hospital doctors are required to report cases of bacillary dysentery to local Center for Disease Control and Prevention. This study included all the bacillary dysentery cases, who were diagnosed by authorized hospitals in Wuhan and reported to Chinese Information System for Diseases Control and Prevention. Daily data on counts of bacillary dysentery cases covering the period 2006–2011 were obtained from Chinese Center for Disease Control and Prevention. In addition to passive surveillance of infectious diseases, the local CDCs conducted active surveillance regularly to reduce the rate of misreporting and underreporting^25^. As bacillary dysentery is a very common disease in study area, it is not difficult for the doctors to make a correct clinical diagnosis. Therefore, it is believed that the disease data quality was reliable^12^.

Daily meteorological data for Wuhan were extracted from the China Meteorological Administration Climatic Dataset Center. The meteorological variables included daily mean temperature (°C), relative humidity (%), rainfall (mm), and wind speed (m/s).

There are 13 districts in Wuhan City. To illustrate the geographic distribution of bacillary dysentery incidence, the district-level polygon map at 1:250,000 scale was obtained from Data Sharing Infrastructure of Earth System Science (www.geodata.cn), on which a district-level layer containing information of each district in Wuhan City was created. For the 6-year period (2006–2011), the annualized average bacillary dysentery incidence was geo-coded and matched to the district-level polygon by administrative codes using the software ArcGIS 9.0^26^. The districts were classified into four categories: low epidemic areas with annual incidence between 0 and 50/100,000, medium epidemic areas with an incidence between 50 and 100/100,000, and high epidemic areas with an incidence higher than 100/100,000.

We used the generalized additive model (GAM) with a log link and allowed Poisson auto-regression and over-dispersion to examine the association between daily meteorological factors and bacillary dysentery occurrence^27^.

Consistent with previous time-series analyses, we controlled for the day of the week (DOW) and public holidays (PH) using categorical indicator variables^28^. We also controlled for long-term and seasonal patterns in daily bacillary dysentery counts using penalized smoothing splines, daily mean temperature, relative humidity, rainfall and air pressure with degrees of freedom (df) based on a priori based on previous studies^29^. Specifically, we used 7 df per year for time trend, 6 df for mean temperature, 3 df for relative humidity, rainfall and wind speed.

We firstly generated the core models for each meteorological variable. Residual plots were used to examine whether there were discernable patterns in the residual of the core models. The core model for temperature can be specified as: 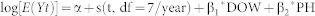
where s() indicates a smoother based on penalized splines, DOW is an indicator for day of week, PH presents a binary variable for the public holiday, and β is the regression coefficient.

After the core models were established. We included the meteorological variable in the model. Because relative humidity and rainfall were highly inter-correlated (correlation coefficient was higher than 0.7), we did not include them in one model.

This infection usually had a short incubation period, one recent study estimated the incubation period to be one day^9^, we investigated the linear effect of various meteorological variables according to different lag days, including the current day (lag0) up to 3 lag days (lag3) and multi-day lags (moving averages for the current day and the previous 1, 2 and 3 days: lag01, lag02, and lag03, respectively). We reported the result as excess risk (ER), defined as the percentage increase in daily Bacillary dysentery for per unit increase in each meteorological factor, with 95% confidence intervals (95% CI).

To testify the linearity assumption of the relationship between the logarithm of daily bacillary dysentery and key meteorological variables, we graphically examined the dose-response curve derived using a smoothing function^30^.

Because the risk estimates usually varied with the model specifications in time-series analysis^24^,^31^, we performed additional sensitivity analyses: use of alternative degrees of freedom (5, 6 and 8 df/year) for temporal adjustment and use of alternative degrees of freedom for various meteorological variables.

All statistical analyses were two-sided and values of P < 0.05 were considered statistically significant. The mgcv package in R software Version 2.14.1 (R Development Core Team, 2012) was used to fit all models and estimate the exact standard errors of regression coefficients.

## Author Contributions

Z.J.L., H.B.S. and H.L.L. conceived, designed and implemented the data analysis, L.G.W., W.G.S., X.X.H., and H.L.L. drafted the manuscript, H.Y.Y., L.N.S., S.X., Q.Z.S., J.S.Z. revised the manuscript.

## Supplementary Material

Supplementary Information Figure s1

## Figures and Tables

**Figure 1:**
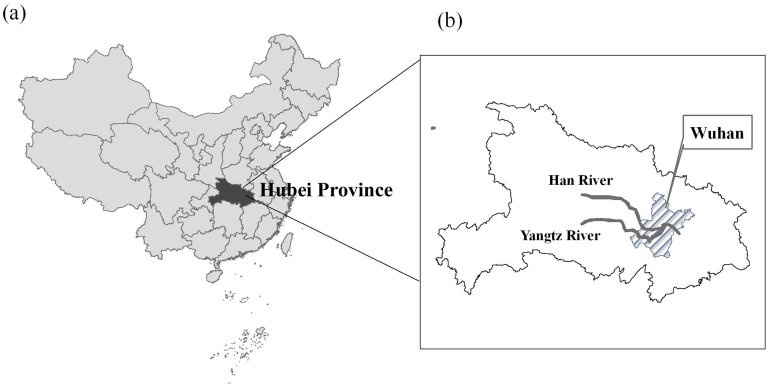
The geographical location of the study area in China ((a) shows the location of Hubei Province in China, (b) is the location of Wuhan in Hubei Province, the map was created with ArcGIS software, 9.0).

**Table 1:** Summary statistics of daily weather conditions and bacillary dysentery in Wuhan, China

Variable	Minimum	Median	Mean	Maximum	SD
Daily cases	0.0	16.0	16.7	47.0	7.6
Temperature (°C)	−2.7	18.8	17.6	35.3	9.5
Relative humidity (%)	21.0	74.0	72.3	100.0	13.0
Rainfall (mm)	0.0	0.0	3.1	197.9	10.6
Wind speed (m/s)	4.0	15.0	16.4	68.0	8.1
