# Supplementary material for: Identifying high-risk areas of bacillary dysentery and associated meteorological factors in Wuhan, China
Source: Sci Rep. 2013 Nov 21;3:3239. doi: 10.1038/srep03239 (PMC3836034; doi:10.1038/srep03239)
Supplement: Supplementary Information — Figure s1 [file srep03239-s1.doc]

Identifying high-risk areas of bacillary dysentery

and associated meteorological factors in Wuhan, China

Zhenjun Li^1,#^, Ligui Wang^2,#^, Weige Sun^3,#^, Xuexin Hou^1^, Haiyan Yang^3^, Lina Sun^1^, Shuai Xu^1^, Qiangzheng Sun^1^, Jingsha Zhang^1^, Hongbin Song^2,*^ & Hualiang Lin^4,*^


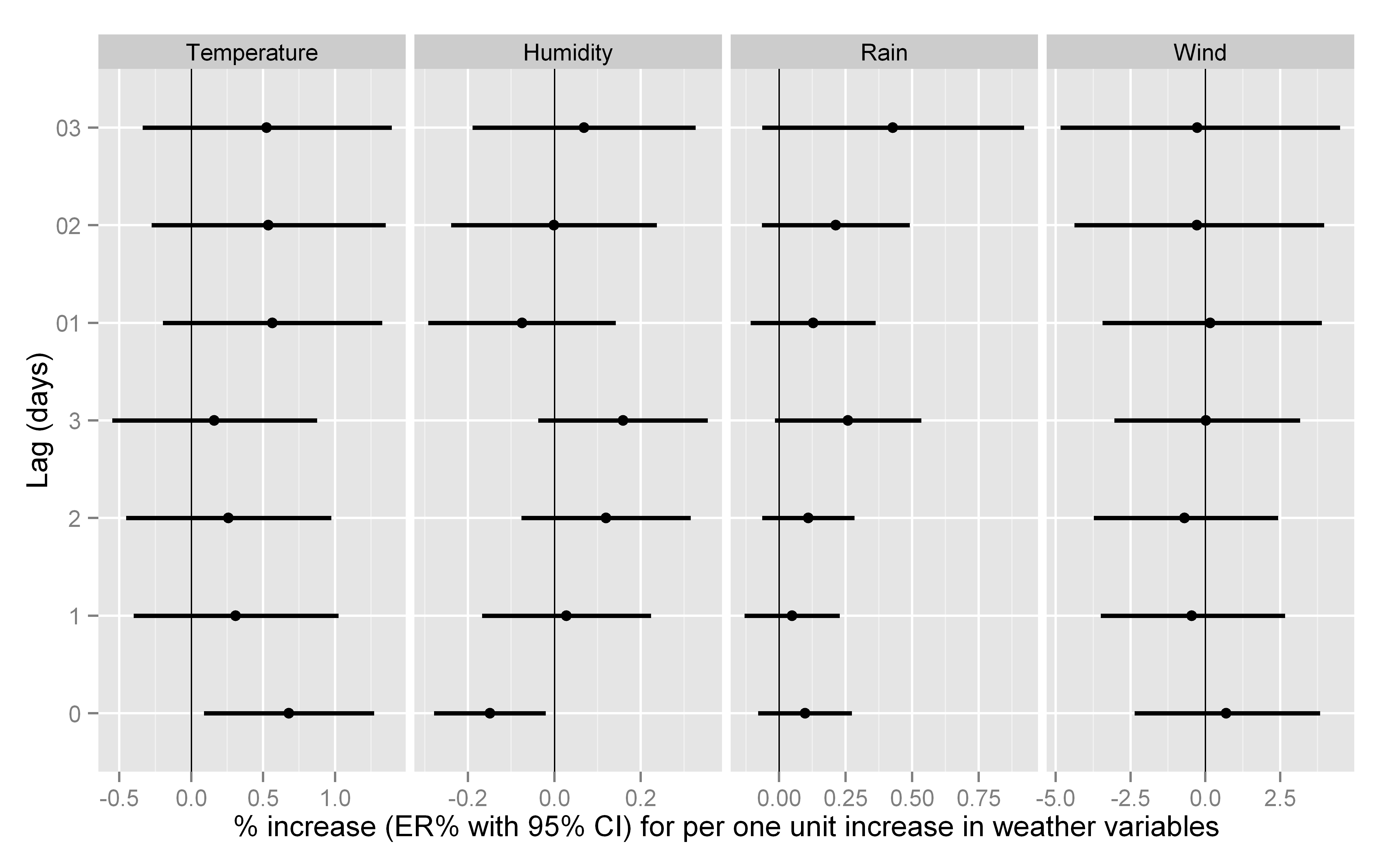


Figure s1 | Excessive risk (ER with 95% CI) in bacillary dysentery in low-risk areas for one unit increase in daily meteorological factors for the current day (lag0) to 3 days before the current day (lag 3) and moving average (lag 01, 02 and 03).
